# Indicators of infertility and fertility care: a systematic scoping review

**DOI:** 10.1093/hropen/hoac047

**Published:** 2022-10-13

**Authors:** Ashraf Nabhan, Mohamed Salama, Mortada Elsayed, Maii Nawara, Menna Kamel, Yasmeen Abuelnaga, Mohanad Ghonim, Farida Elshafeey, Rana Abdelhadi, Sara Gebril, Shahd Mahdy, Dana Sarhan, Gitau Mburu, James Kiarie

**Affiliations:** Department of Obstetrics and Gynecology, Faculty of Medicine, Ain Shams University, Cairo, Egypt; Egyptian Center for Evidence Based Medicine, Cairo, Egypt; Department of Obstetrics and Gynecology, Faculty of Medicine, Ain Shams University, Cairo, Egypt; Department of Obstetrics and Gynecology, Faculty of Medicine, Ain Shams University, Cairo, Egypt; Department of Obstetrics and Gynecology, Faculty of Medicine, Ain Shams University, Cairo, Egypt; Egyptian Center for Evidence Based Medicine, Cairo, Egypt; Egyptian Center for Evidence Based Medicine, Cairo, Egypt; Egyptian Center for Evidence Based Medicine, Cairo, Egypt; Egyptian Center for Evidence Based Medicine, Cairo, Egypt; Egyptian Center for Evidence Based Medicine, Cairo, Egypt; Egyptian Center for Evidence Based Medicine, Cairo, Egypt; Egyptian Center for Evidence Based Medicine, Cairo, Egypt; Egyptian Center for Evidence Based Medicine, Cairo, Egypt; The UNDP/UNFPA/UNICEF/WHO/World Bank Special Programme of Research, Development and Research Training in Human Reproduction (HRP Research), Geneva, Switzerland

**Keywords:** quality indicators, assisted reproduction, infertility, metrics, fertility care

## Abstract

**STUDY QUESTION:**

What is the scope of literature regarding infertility and fertility care indicators in terms of types and dimensions of these indicators?

**SUMMARY ANSWER:**

Most available infertility and fertility care indicators are outcomes indicators of effectiveness and efficiency dimensions.

**WHAT IS KNOWN ALREADY:**

The use of appropriate, relevant and valid indicators of infertility and fertility care is critical for monitoring access, equity and utilization.

**STUDY DESIGN, SIZE, DURATION:**

A systematic scoping review was conducted. We searched MEDLINE, Pubmed, JSTOR, CINAHL, Web of Science and Scopus electronic databases from inception to May 2022 without imposing language or date restrictions. We searched gray literature and online libraries of relevant organizations. We hand-searched the list of relevant references.

**PARTICIPANTS/MATERIALS, SETTING, METHODS:**

This scoping systematic review followed the framework of Arksey and O’Malley and the Joanna Briggs Institute guidelines. Records identified by the search were independently screened and data were extracted. We performed conceptual synthesis by grouping the reported indicators by typology and dimensions. Structured tabulation and graphical synthesis were used along with narrative commentary.

**MAIN RESULTS AND THE ROLE OF CHANCE:**

We included 46 reports from 88 countries. The reporting of infertility and fertility care indicators was voluntary in 63 countries (72%) and compulsory in 25 countries (28%). Reporting for cycles or deliveries was based on individual cycles in 56 countries (64%) and on cumulative cycles in 32 countries (36%). Most indicators were utilized as outcome indicators with fewer being process indicators or structural indicators. For the dimension of indicators, most indicators were utilized as effectiveness and efficiency indicators with fewer utilized as indicators of safety, patient-centeredness, equity and timeliness.

**LIMITATIONS, REASONS FOR CAUTION:**

Most indicators fall into the domain of assisted reproductive technology and are reported by fertility clinics. Indicators of safety, patient-centeredness, equity and timeliness as well as non-clinical indicators are almost invisible.

**WIDER IMPLICATIONS OF THE FINDINGS:**

A wide range of indicators of infertility and fertility care exist in literature. Most indicators were effectiveness and efficiency indicators, while indicators of safety, patient-centeredness, equity and timeliness remain almost invisible. The scope of the current indicators indicates a predominant focus on clinical metrics, with substantial invisibility of non-clinical indicators and indicators outside the ART domain. These gaps need to be considered in further work of identifying a core set of indicators.

**STUDY FUNDING/COMPETING INTEREST(S):**

This work received funding from the UNDP-UNFPA-UNICEF-WHO-World Bank Special Programme of Research, Development and Research Training in Human Reproduction (HRP), a cosponsored program executed by the World Health Organization (WHO). The authors had no competing interests.

**TRIAL REGISTRATION NUMBER:**

Open Science Framework vsu42.

WHAT DOES THIS MEAN FOR PATIENTS?

This study provides an overview of the available ways to assess different aspects of infertility and fertility care. Current indicators of the provision of care focus mainly on clinical endpoints of effectiveness, with minimal availability of non-clinical aspects of fertility care. Safety of care and equitable access to care were almost invisible. The success of treatment (for example the achievement of pregnancy) is often considered the gold standard for assessing the quality of fertility care and treatment of infertility. Although recipients of care also value the ability of the care to succeed, it is essential to have indicators that reflect equitable access and the ability to minimize potential harm. Similarly, other indicators are extremely important to determine how efficient, patient-centered and timely the care is. Organizations can utilize available indicators that best suit their patients’ needs. The results of this review serve as the basis to develop a core set of indicators using a process that involves all relevant stakeholders.

## Introduction

Infertility is a major public health issue globally, affecting 8–12% of individuals in reproductive age. Approximately 48 million couples and 186 million individuals live with infertility (Mascarenhas *et al.*, 2012). Infertility is particularly high in sub-Saharan Africa, south and central Asia, North Africa and the Middle East, as well as Central and Eastern Europe (Boivin *et al.*, 2007; Mascarenhas *et al.*, 2012; Inhorn and Patrizio, 2015; Vander Borght and Wyns, 2018; World Health Organization, 2020).

Provision of safe fertility care is central to the achievement of Sustainable Development Goal (SDG) 3 to ensure healthy lives and promote well-being for all at all ages, and SDG 5, to achieve gender equality and empower all women and girls. Responding to the needs of people with infertility also plays an integral role in ensuring universal access to healthcare. The World Health Organization (WHO) recognizes infertility as an essential component of sexual and reproductive health, however, the availability, access to and quality of infertility services might be inequitable (Ombelet, 2011). Areas of the world with the highest rates of infertility are often those with poor access to infertility services including assisted reproductive technology (ART) (World Health Organization, 2004, 2020; Inhorn and Patrizio, 2015).

Having the right compendium of government policy is essential in increasing access to fertility care as part of universal health coverage. Once fertility policies are in place, it is essential to ensure that their implementation is monitored, and the quality of services is continually improved (World Health Organization, 2020). To this end, it is essential to establish robust indicators of infertility and the provision of infertility services. Indicators are important markers of health status, service provision and resource availability, and are often designed to enable the monitoring of service performance and overall program goals (World Health Organization, 2006).

Currently, one indicator is being used to monitor progress in relation to infertility globally, that is, prevalence. However, the growing need for and rapid evolution of fertility care services including medically assisted reproduction warrants an expanded set of indicators related to infertility and fertility care at both global and national levels (Dancet *et al.*, 2013). This is particularly relevant given that indicators determine resource allocation, implementation, monitoring and accountability, both globally and nationally (World Health Organization, 2006). Different groups have developed sets of indicators either for practice or research (Duffy *et al.*, 2021).

To inform the selection of potential indicators, WHO undertook two reviews: one review, on the prevalence of infertility and different methods to estimate it, and a second review, to identify potential indicators for infertility and fertility care that complement prevalence. The identification of appropriate, relevant and valid indicators will facilitate effective monitoring of progress in fertility care access, equity, utilization and impact.

Specifically, the objective of this scoping review was to map the published literature related to indicators of infertility and fertility care and their types and dimensions.

## Materials and methods

Given the broad and complex concept of indicators, and the anticipation that the literature would include studies with different methodologies, a scoping review approach was utilized as the most appropriate synthesis method to map the range, breadth and extent of literature regarding indicators of infertility and fertility care.

The scoping review was guided by a framework originally proposed by Arksey and O’Malley (2005), which was subsequently improved by Levac *et al.* (2010), Colquhoun *et al.* (2014) and the Joanna Briggs Institute guidelines (Peters *et al.*, 2015). In addition, we report the review following the Preferred Reporting Items for Systematic Review and Meta-Analysis: extension for scoping review (Tricco *et al.*, 2018). The review has been registered in the Open Science Framework platform (osf.io/vsu42).

The process of performing this review involved the following stages.

### Stage 1: defining research questions

The following questions guided the scoping review. What are the indicators of fertility care? What are the types and dimensions of these indicators? What are the reported methods for measuring these indicators? What is the map of the countries, by region, with available data? What entities are responsible for compiling data in these countries?

### Stage 2: identifying relevant studies

A systematic search was conducted in May 2021 and updated in May 2022 in three steps to identify both published and unpublished primary sources as well as reviews. In the first step, we conducted an initial limited search of one bibliographic database and analyzed the text words contained in the title and abstract of retrieved papers, as well as the index terms used to describe the articles. In the second step, we identified text words and index terms that were used to develop the search strategy. This was further refined through team discussion. The strategy for searching bibliographic databases included the following terms Humans[Mesh] AND (Infertility[Mesh] OR assisted reproduct* [tw] OR infertility [tw] OR subfertility [tw]) AND (Patient Care/standards [Mesh] OR Health Care Quality Indicators [Mesh] OR indicator* [tw]). The search strategy for different databases can be found in Supplementary Table SI. We searched MEDLINE, PubMed, CINAHL, Web of Science, JSTOR and Scopus electronic databases.

Given the nature of this review, gray literature was also searched. This included online libraries of relevant organizations including World Health Organizations (WHO), United Nations Population Fund (UNFPA), International Federation of Gynecology and Obstetrics (FIGO), International Federation of Fertility Societies (IFFS), International Committee for Monitoring Assisted Reproductive Technologies (ICMART) and the Demographic Health Survey (DHS) Program. We also searched reports and proceedings of conferences from the following organizations: European Society of Human Reproduction and Embryology (ESHRE), the American Society for Reproductive Medicine (ASRM), Latin American Network of Assisted Reproduction (REDLARA), Asia Pacific Initiative on Reproduction (ASPIRE), The African Network and Registry for Assisted Reproductive Technology (ANARA), Human Fertilization and Embryology Authority (HFEA), Canadian Fertility and Andrology Society (CFAS), The Australia and New Zealand Assisted Reproduction Database (ANZARD) and the CDC National ART Surveillance System (NASS). Finally, we hand-searched the list of references of relevant articles and explored the citations by logs of relevant articles. We did not restrict it by date nor language.

### Stage 3: study selection

#### Inclusion criteria

We included reports of population (individuals or couples with infertility or seeking fertility care), concept (indicators of infertility and fertility care, with infertility defined as the failure to achieve pregnancy after one year of unprotected and regular sexual intercourse or because of an impaired capacity of a person to reproduce either as an individual or with the partner, and fertility care defined as the spectrum of healthcare services to prevent, diagnose and treat infertility; Zegers-Hochschild *et al.*, 2017), context (all levels and types of health service), and types of studies (all designs including primary and secondary research).

#### Selection of studies

All records identified by the search were independently screened by two authors (M.K. and F.E.) based on the titles and abstracts. The second stage of selection was based on reviewing the full text of potentially relevant articles. If an agreement regarding abstract or full article inclusion could not be reached between the two reviewers, an opinion was requested from a third reviewer (A.N.).

### Stage 4: data charting process

An electronic data-charting form was used to extract the data items outlined in Supplementary Table SII. Given the broad scope of the review, the authors worked in two independent groups (M.K., F.E. reporting for each group) to extract data, continuously updating the data-charting form in an iterative process. For each included study, the methodological quality was assessed independently by two reviewers, using the corresponding Mixed-Methods Appraisal Tool criteria (Hong *et al.*, 2018). We included all studies regardless of their quality because the aim was to assess the extent of the available literature.

### Stage 5: collating, summarizing and reporting results

Two analytic frameworks were used to organize the full list of mapped indicators. These were the Donabedian framework for the types of indicators (framework components: structure, process, outcomes) (Table I) (Donabedian, 1988) and the Institute of Medicine (IOM) framework for the dimensions of indicators (framework components: safety, effectiveness, patient-centeredness, timeliness, efficiency, equity) (Table II) (Institute of Medicine. Committee on Quality of Health Care in America, 2001).

**Table I:** Definitions of the types of indicators.

Type	Definition
Structural	Structural indicators describe the type and amount of resources used by a health system or organization to deliver programs and services, and they relate to the presence or number of staff, money, beds, supplies and buildings.
Process	Process indicators reflect generally accepted recommendations for clinical practice. Processes are a series of inter-related activities undertaken to achieve objectives, and thus process indicators assess what care providers do for the patient and how well it is done.
Outcome	Outcome indicators reflect the impact of the health care service on the health status of patients.

**Table II:** Definitions of the dimensions of indicators.

Dimension	Definition
Safe	Avoiding harm to patients from the care that is intended to help them.
Effective	Providing services based on scientific knowledge to all who could benefit and refraining from providing services to those not likely to benefit patients (i.e. avoiding underuse and misuse, respectively).
Patient-centered	Providing care that is respectful of and responsive to individual patient preferences, needs and values, and ensuring that patient values guide all clinical decisions.
Timely	Reducing waiting time and sometimes harmful delays for both those who receive and those who give care.
Efficient	Avoiding waste, including waste of equipment, supplies, ideas and energy.
Equitable	Providing care that does not vary in quality because of the personal characteristics of the patients, such as gender, ethnicity, geographic location and socioeconomic status.

### Stage 6: consultation exercise

In this step, stakeholders outside the study review team were invited to provide their insights to inform and validate findings from the scoping review. WHO identified and invited a range of stakeholders to participate in a technical consultation, the preliminary results of the scoping review (Stages 1–5 above) were prepared as background summary documents and shared with participants in advance of the technical consultation. Participants of the technical consultation were selected purposively to achieve broad representation from different global regions, and to ensure a range of perspectives were captured from different experts. Participants included clinicians, ministry of health officials, psychologists, community advocates of people with infertility, demographers, statisticians and epidemiologists with an interest in the measurement of infertility and fertility care. Specifically, the experts were asked to provide feedback on the preliminary results of the review, based on the following questions. Are the identified indicators relevant and feasible and do they form a good starting point for further prioritization? Are there additional relevant indicators that should be captured for further discussion in addition to the indicators presented above, and if so, which? Is there an additional dimension (apart from what is presented in the summary tables) that should be included in the summaries for each indicator? Why? Are there additional agencies that should be responsible for collecting the indicators? Are there additional considerations that should be considered in prioritizing the most appropriate fertility service indicators? What are they?

## Results

### Literature search results

**Figure 1.**
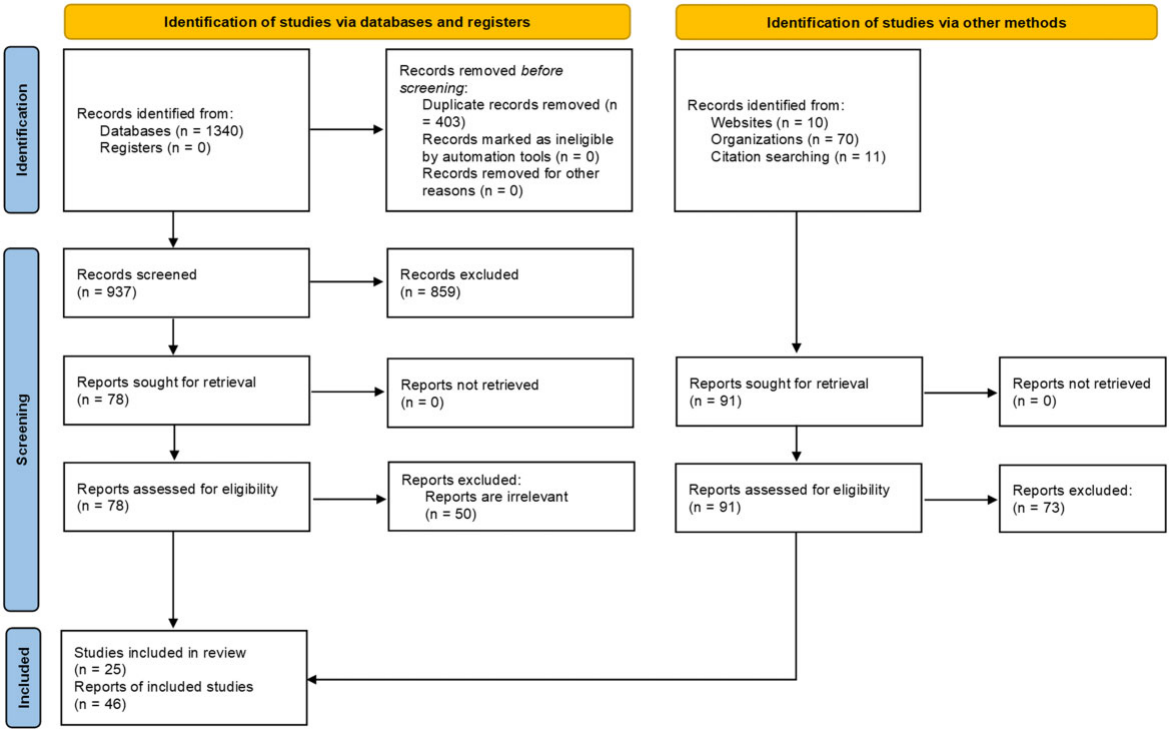
**PRISMA flowchart.** PRISMA, Preferred Reporting Items for Systematic Reviews and Meta-Analyses, Extension for Scoping Reviews.

The electronic search yielded 1340 records, while an additional 91 records were identified from other sources. Following the removal of duplicates and screening of titles and abstracts, 169 full text reports were assessed for inclusion. Finally, 46 reports were included in this scoping review as depicted in the PRISMA flowchart (Fig. 1).

These reports included data from 88 countries (38 countries in Europe, 20 countries in Africa, 16 countries in Latin America and the Caribbean, 10 countries in Asia, two countries in Oceania and two countries in North America) (Table III).

**Table III:** Mapping of reporting of indicators.

Region	Country	Responsibility	Reporting methods: cycles	Reporting methods: deliveries	Completeness	Requirement
Africa						
	Benin	Fertility clinics	IPD	IPD	Partial	Voluntary
	Burkina Faso	Fertility clinics	IPD	IPD	Partial	Voluntary
	Cameroon	Fertility clinics	IPD	IPD	Partial	Voluntary
	Egypt	Fertility clinics	IPD	IPD	Partial	Voluntary
	Ethiopia	Fertility clinics	IPD	IPD	Partial	Voluntary
	Ghana	Fertility clinics	IPD	IPD	Partial	Voluntary
	Ivory Coast	Fertility clinics	IPD	IPD	Partial	Voluntary
	Kenya	Fertility clinics	IPD	IPD	Partial	Voluntary
	Libya	Fertility clinics	IPD	IPD	Partial	Voluntary
	Mali	Fertility clinics	IPD	IPD	Partial	Voluntary
	Mauritius	Fertility clinics	IPD	IPD	Partial	Voluntary
	Morocco	Fertility clinics	IPD	IPD	Partial	Voluntary
	Nigeria	Fertility clinics	IPD	IPD	Partial	Voluntary
	Senegal	Fertility clinics	IPD	IPD	Partial	Voluntary
	South Africa	Fertility clinics	IPD	IPD	Partial	Voluntary
	Sudan	Fertility clinics	IPD	IPD	Partial	Voluntary
	Togo	Fertility clinics	IPD	IPD	Partial	Voluntary
	Tunisia	Fertility clinics	IPD	IPD	Partial	Voluntary
	Uganda	Fertility clinics	IPD	IPD	Partial	Voluntary
	Zimbabwe	Fertility clinics	IPD	IPD	Partial	Voluntary
Asia						
	Bangladesh	Fertility clinics	Aggregate	Aggregate	Partial	Voluntary
	China	National Health Authority	IPD	IPD	All	Voluntary
	India	Fertility clinics	Aggregate	Aggregate	Partial	Compulsory
	Indonesia	Fertility clinics	Aggregate	Aggregate	Partial	Voluntary
	Myanmar	Fertility clinics	Aggregate	Aggregate	Partial	Voluntary
	Singapore	Fertility clinics	Aggregate	Aggregate	Partial	Voluntary
	Taiwan	Fertility clinics	Aggregate	Aggregate	Partial	Voluntary
	Kazakhstan	Medical Organization	Aggregate	Aggregate	Partial	Voluntary
	Armenia	Professional society	Aggregate	Aggregate	Partial	Voluntary
	Japan	Professional society	IPD	IPD	Partial	Compulsory
Europe						
	Belarus	Medical Organization	Aggregate	Aggregate	Partial	Voluntary
	Germany	Medical Organization	IPD	IPD	Partial	Voluntary
	Moldova	Medical Organization	Aggregate	Aggregate	Partial	Voluntary
	Netherlands	Medical Organization	Aggregate	Aggregate	All	Compulsory
	Poland	Medical Organization	Aggregate	Aggregate	All	Voluntary
	Russia	Medical Organization	Aggregate	Aggregate	Partial	Voluntary
	Serbia	Medical Organization	Aggregate	Aggregate	Partial	Voluntary
	Sweden	Medical Organization	IPD	IPD	Partial	Voluntary
	Switzerland	Medical Organization	IPD	IPD	All	Voluntary
	Austria	National Health Authority	IPD	IPD	All	Compulsory
	Belgium	National Health Authority	IPD	IPD	All	Compulsory
	Bulgaria	National Health Authority	Aggregate	Aggregate	All	Compulsory
	Cyprus	National Health Authority	Aggregate	Aggregate	All	Voluntary
	Czech Republic	National Health Authority	IPD	IPD	Partial	Compulsory
	Denmark	National Health Authority	IPD	IPD	All	Compulsory
	Estonia	National Health Authority	Aggregate	Aggregate	All	Compulsory
	Finland	National Health Authority	Aggregate	Aggregate	All	Compulsory
	France	National Health Authority	IPD	IPD	All	Compulsory
	Greece	National Health Authority	Aggregate	Aggregate	All	Compulsory
	Hungary	National Health Authority	IPD	IPD	Partial	Compulsory
	Iceland	National Health Authority	Aggregate	Aggregate	All	Compulsory
	Italy	National Health Authority	Aggregate	Aggregate	All	Compulsory
	Malta	National Health Authority	IPD	IPD	All	Compulsory
	Norway	National Health Authority	Aggregate	Aggregate	All	Compulsory
	Portugal	National Health Authority	IPD	IPD	All	Compulsory
	Republic of North Macedonia	National Health Authority	Aggregate	Aggregate	Partial	Voluntary
	Romania	National Health Authority	Aggregate	Aggregate	Partial	Compulsory
	Slovenia	National Health Authority	Aggregate	Aggregate	All	Compulsory
	Spain	National Health Authority	Aggregate	Aggregate	Partial	Compulsory
	United Kingdom	National Health Authority	IPD	IPD	All	Compulsory
	Albania	Personal initiative	IPD	IPD	Partial	Voluntary
	Bosnia and Herzegovina	Personal initiative	Aggregate	Aggregate	Partial	Voluntary
	Latvia	Personal initiative	Aggregate	Aggregate	Partial	Voluntary
	Lithuania	Personal initiative	Aggregate	Aggregate	Partial	Voluntary
	Luxembourg	Personal initiative	Aggregate	Aggregate	All	Compulsory
	Montenegro	Personal initiative	Aggregate	Aggregate	Partial	Voluntary
	Ireland	Professional society	IPD	IPD	Partial	Voluntary
	Ukraine	Professional society	Aggregate	Aggregate	Partial	Voluntary
Latin America and the Caribbean						
	Argentina	Fertility clinics	IPD	IPD	Partial	Voluntary
	Bolivia	Fertility clinics	IPD	IPD	Partial	Voluntary
	Brazil	Fertility clinics	IPD	IPD	Partial	Voluntary
	Chile	Fertility clinics	IPD	IPD	Partial	Voluntary
	Colombia	Fertility clinics	IPD	IPD	Partial	Voluntary
	Costa Rica	Fertility clinics	IPD	IPD	Partial	Voluntary
	Dominican Republic	Fertility clinics	IPD	IPD	Partial	Voluntary
	Ecuador	Fertility clinics	IPD	IPD	Partial	Voluntary
	Guatemala	Fertility clinics	IPD	IPD	Partial	Voluntary
	Mexico	Fertility clinics	IPD	IPD	Partial	Voluntary
	Nicaragua	Fertility clinics	IPD	IPD	Partial	Voluntary
	Panama	Fertility clinics	IPD	IPD	Partial	Voluntary
	Paraguay	Fertility clinics	IPD	IPD	Partial	Voluntary
	Peru	Fertility clinics	IPD	IPD	Partial	Voluntary
	Uruguay	Fertility clinics	IPD	IPD	Partial	Voluntary
	Venezuela	Fertility clinics	IPD	IPD	Partial	Voluntary
	United States	National Health Authority	IPD	IPD	All	Compulsory
	Canada	Professional society	IPD	IPD	All	Voluntary
Oceania						
	Australia	Professional society	IPD	IPD	All	Compulsory
	New Zealand	Professional society	IPD	IPD	All	Compulsory

IPD: individual cycles; Aggregate: summaries of cycles reported by the clinics.

The included studies were primarily observational studies (38/46), in addition to eight narrative reviews. Most of the primary studies (31/38) were reports of routinely collected data. In the assessment of the quality of included primary reports, we judged most reports to be of high quality, although there were some concerns related to the incomplete reporting of data from fertility clinics (Supplementary Table SIII).

### Typology and dimensions of indicators

**Figure 2.**
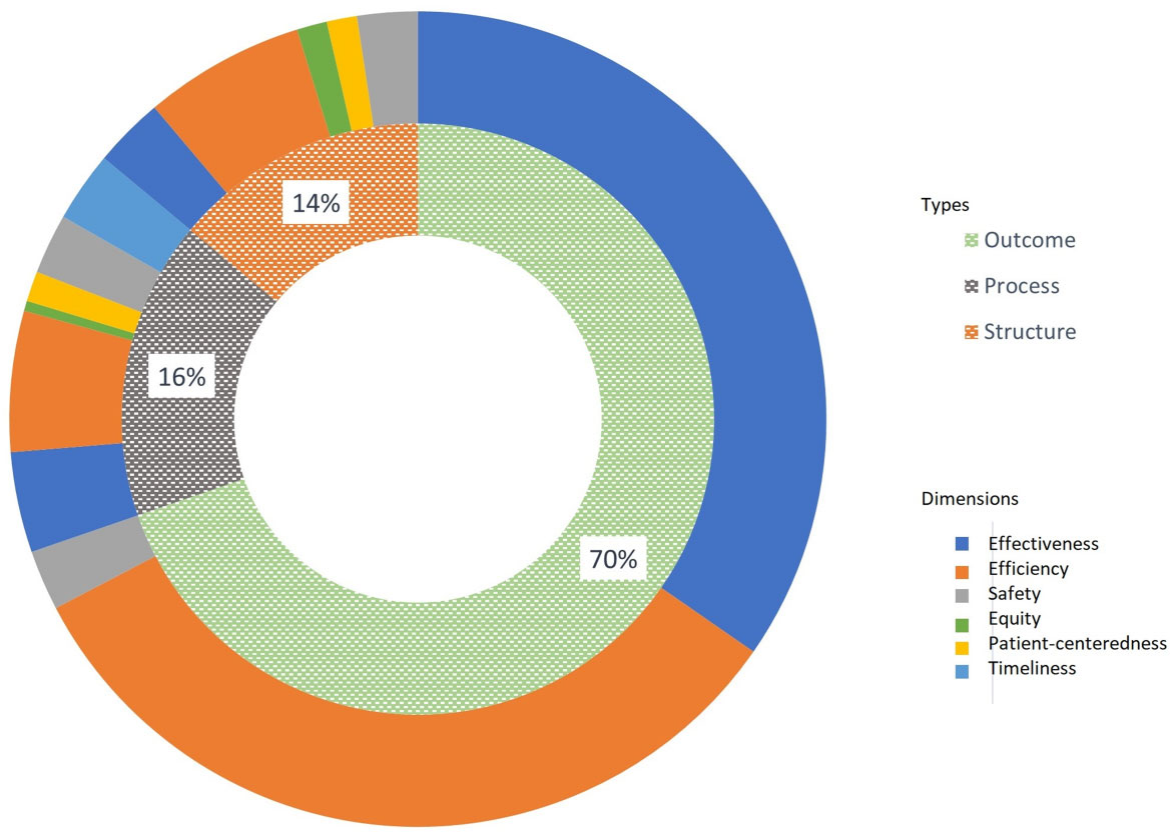
**Doughnut chart of the breadth of reported infertility indicators by types and dimensions.** The relative abundance of literature is shown in the inner doughnut for the types (outcome, process and structure) and in the outer doughnut for the dimensions (effectiveness, efficiency, equity, patient-centeredness, safety and timeliness) of indicators.

We identified 147 specific indicators. These indicators were conceptually grouped using the pre-specified analytical frameworks: by typology (structural, process, outcome) and by dimensions (safety, effectiveness, patient-centeredness, timeliness, efficiency, equity) (Davies *et al.*, 2004; Rutstein and Shah, 2004; Germond *et al.*, 2008; Mourad *et al.*, 2008; Breejen *et al.*, 2013; Dancet *et al.*, 2013; Malhotra *et al.*, 2013; Wilkinson *et al.*, 2017; Fauser, 2019; Jain *et al.*, 2019; Zahmatkeshan *et al.*, 2019a,b; Bai *et al.*, 2020; Centers for Disease Control and Prevention, 2020; De Geyter *et al.*, 2020; Dyer *et al.*, 2020a,b; Fischer and Scott, 2020; Lanes *et al.*, 2020; Newman *et al.*, 2020; Pirtea *et al.*, 2020; Sunderam *et al.*, 2020; Zegers-Hochschild *et al.*, 2020). The classification of indicators by typology and dimensions is shown in Fig. 2.

A matrix of all available indicators classified by typology and dimensions was developed. The matrix shows that most indicators fall into the domain of assisted reproductive technology and that 104 indicators were used across two dimensions of the same typology (Table IV).

**Table IV:** Infertility and fertility care indicators organized by type and dimension.

Typology	Indicator	Effectiveness	Efficiency	Equity	Patient-centeredness	Safety	Timeliness
Outcome	1PN rate (ICSI)	X	X				
	1PN rate (IVF)	X	X				
	Achievement of the minimum standards for the KPIs established for monitoring laboratory performance by consensus papers (e.g. Vienna consensus KPIs for fresh IVF and ICSI cycles or Alpha KPIs for oocyte and embryo cryopreservation) per month	X	X				
	Average number of intended retrievals per new patient	X	X				
	Average number of transfers per intended retrieval	X	X				
	Blastocyst development rate	X	X				
	C-LBR	X	X				
	Cleavage rate	X	X				
	Cleavage rate for ICSI-fertilized cryopreserved oocytes.	X	X				
	CPR	X	X				
	Cumulative ART success rates	X	X				
	Day 2 embryo development rate	X	X				
	Day 3 embryo development rate	X	X				
	Day-5 blastulation rate	X	X				
	Drugs for stimulation: GnRH agonist, GnRH antagonist, clomiphene citrate	X	X				
	Oocyte donations from the related/known donors	X	X				
	Embryo morphology data	X	X				
	Embryos donated for research	X	X				
	ET—Difficult transfers	X	X				
	ET—Retained embryos	X	X				
	Failed fertilization rate (IVF)	X	X				
	Fertilization rate	X	X				
	Fertilization rate of cryopreserved oocytes by ICSI	X	X				
	Good blastocyst development rate	X	X				
	ICSI damage rate	X	X				
	ICSI normal fertilization rate	X	X				
	Implantation rate (blastocyst stage)	X	X				
	Implantation rate (cleavage stage)	X	X				
	Implantation rate for cryopreserved blastocysts (women <38 years).	X	X				
	Implantation rate for post-cryopreservation embryos (women <38 years).	X	X				
	IVF normal fertilization rate	X	X				
	IVF polyspermy rate	X	X				
	LBR	X	X				
	MII oocytes rate	X	X				
	Miscarriage rate	X	X				
	Number of double embryo transfer in ED cycles	X	X				
	Number of oocytes shared between the donor and the recipient (anonymous or related)	X	X				
	Number of oocytes shared between the donor and the recipient	X	X				
	Number of intended retrievals per live birth	X	X				
	Number of oocytes	X	X				
	OPR	X	X				
	OPU % Hemorrhage					X	
	OPU % Oocytes retrieved/mature follicles	X	X				
	OPU % Other complication requiring hospitalization					X	
	OPU % Pelvic infection					X	
	OS % Canceled retrieval	X	X				
	OS % OHSS					X	
	OS % Retrieval failure (no oocytes)	X	X				
	Percentage of retrievals resulting in singleton live births	X	X				
	Percentage of cycles canceled prior to retrieval or thaw	X	X				
	Percentage of cycles for fertility preservation	X	X				
	Percentage of cycles stopped between retrieval and transfer or banking	X	X				
	Percentage of intended retrievals resulting in live births	X	X				
	Percentage of intended retrievals resulting in singleton live births	X	X				
	Percentage of new patients having live births after 1 intended retrieval	X	X				
	Percentage of new patients having live births after 1 or 2 intended retrievals	X	X				
	Percentage of new patients having live births after all intended retrievals	X	X				
	Percentage of retrievals resulting in live births	X	X				
	Percentage of transfers of at least one embryo with ICSI	X	X				
	Percentage of transfers of at least one embryo with PGT	X	X				
	Percentage of transfers resulting in live births	X	X				
	Percentage of transfers resulting in singleton live births	X	X				
	Percentage of transfers using a gestational carrier	X	X				
	Percentage of transfers using frozen embryos	X	X				
	Proportion of ART births among all births	X					
	Proportion of blastocysts that are more-or-less intact post-cryopreservation.	X	X				
	Proportion of blastocysts that re-expand within 3 h post-cryopreservation.	X	X				
	Proportion of early blastocysts post-cryopreservation that expand during overnight culture prior to embryo transfer.	X	X				
	Proportion of embryos with ≥50% blastomeres intact post-cryopreservation.	X	X				
	Proportion of embryos with all blastomeres intact post-cryopreservation.	X	X				
	Proportion of MII oocytes at ICSI	X	X				
	Proportion of oocyte retrieval cycles that have blastocysts suitable for freezing.	X	X				
	Proportion of oocyte retrieval cycles that have embryos suitable for freezing.	X	X				
	Proportion of oocyte retrieval cycles that have zygotes for freezing.	X	X				
	Proportion of oocytes recovered	X	X				
	Proportion of oocytes that are intact post-cryopreservation.	X	X				
	Proportion of post-cryopreservation embryos that cleave during overnight culture.	X	X				
	Proportion of post-cryopreservation zygotes that cleave during overnight culture.	X	X				
	Proportion of zygotes that appear intact post-cryopreservation.	X	X				
	SIR	X	X				
	Sperm motility post-preparation	X	X				
	Success rates for art transfers among patients using oocytes or embryos from a donor: percentage of transfers resulting in live births	X	X				
	Success rates for art transfers among patients using oocytes or embryos from a donor: percentage of transfers resulting in singleton live births	X	X				
	Successful biopsy rate	X	X				
	Thawing survival	X	X				
	The number of fresh ART cycles with complications (OHSS, hemorrhage, infection) as a result of MAR relative to the total number of fresh ART cycles during a certain time period					X	
	The number of fresh ART cycles with severe complications (OHSS, bleeding, infection, complaints of serious pain) resulting from the fertility treatment, which require hospitalization relative to the total number of fresh ART cycles during a certain time period					X	
	The number of live births (the complete expulsion or extraction of a product of fertilization that shows evidence of life) after a fresh ART cycle with embryo transfer relative to the total number of fresh ART cycles with embryo transfer during a certain time period	X					
	The number of patients who after a maximum of three fresh ART cycles (oocyte aspiration actually performed) had a live birth (the expulsion or extraction of minimally one fetus showing evidence of life) relative to the total number of patients starting an ART cycle during a certain time period	X					
	The number of pregnancies in women younger than 36 years old as a result of a fresh ART cycle relative to the total amount of fresh ART cycles in women younger than 36 years old during a certain time period	X					
	The number of treated patients who go home with a live born baby relative to the total number of treated patients during a certain time period	X					
	Total service volume of IVF/ICSI/FET/PGD	X	X				
	Twin PR	X	X				
Process	Average duration of gamete/embryo manipulations in minutes		X				X
	Gonadotropins—Total dose	X	X				
	Gonadotropins—Type	X	X				
	Interoperator agreement in oocyte/embryo morphological grading	X	X				
	Interval between the scheduled and the effective time for a given procedure		X				X
	Intrauterine insemination: IUI-H, IUI-D	X	X				
	Number of accidents (e.g. gamete/embryo loss during denudation) per operator relative to the total number of ART procedures conducted in a given time period	X				X	
	Number of ART clinics reporting data on their ART services		X				
	Number of identified mistakes (e.g. mislabeled samples) per operator relative to the total number of ART procedures conducted in a given time period	X				X	
	Percentage of laboratory staff injuries while handling liquid nitrogen per number of ART procedures per year					X	
	Proportion of fragments lysed or lost after embryo biopsy	X	X				
	Reason for Using ART		X		X		
	The average duration of the waiting time during MAR per patient between having the need for and attending an urgent consultation in case of unexpected negative results (e.g. fertilization failure) during a certain time period						X
	The average duration of the waiting time in the waiting room per patient between the agreed time to start a consultation and actual starting time of the consultation during a certain time period						X
	The average duration of the waiting time per new patient between the asking and the getting of the first appointment during a certain time period						X
	The average duration of the waiting time per patient between the first appointment and the start of the first treatment cycle during a certain time period						X
	The number of cross-contamination or operator infections per number of ART procedures conducted with infectious material					X	
	The number of MAR cycles in which gametes or embryos get lost as a result of an accident, human error or mistake relative to the total number of MAR cycles during a certain time period					X	
	The number of patients of a fertility clinic to whom psychosocial counseling was offered relative to the total number of patients of that fertility clinic during a certain time period				X		
	The number of patients undergoing a very thorough diagnostic phase and reaching a diagnosis prior to starting MAR relative to the total number of patients starting MAR during a certain time period		X				
	The number of patients who opinionated that she/he is being respected by her/his physician relative to the total number of interrogated patients during a certain time period			X			
	The number of patients who opinionated that their personal experiences and wishes were actually heard relative to the total number of interrogated patients during a certain time period				X		
	The number of reported mistakes or incidents caused by all care providers relative to the number of treatment cycles during a certain time period					X	
	The percentage of donor oocyte recipients of more than 55 years of age	X	X				
	Time elapsed between drop deposition and oil coverage during dish preparation		X				X
	Type of cycle in relation to: oocytes, fresh, non-donor, fresh, donor cycle, sharing cycle, thaw cycle non-donor, thaw cycle donor, fresh recipient cycle	X	X				
	Type of cycle: long, short, natural, substituted, other	X	X				
Structural	Achievement of competency values established by consensus papers (e.g. Vienna consensus KPIs for fresh IVF and ICSI cycles or Alpha KPIs for oocyte and embryo cryopreservation) per operator per month	X	X				
	Average time required to move a dish or sample from a place to another	X				X	
	Clinic Current Services		X		X		
	Ever-use of any infertility services		X				
	Number of approved clinics providing ART services		X				
	Number of ART cycles being performed		X				
	Number of ART cycles per million population per year		X				
	Number of clinics providing cryopreservation		X				
	Number of clinics providing fertility preservation		X				
	Number of clinics providing GIFT cycles		X				
	Number of clinics providing ICSI		X				
	Number of clinics providing PGT (PGD/PGS) services		X				
	Number of CPD credits per operator per year	X	X				
	Number of critical instruments (e.g. incubators, safety cabinets, micromanipulators) relative to the total number of ART procedures conducted in a given time period	X				X	
	Number of operators relative to the total number of ART procedures conducted in a given time period	X				X	
	Number of unscheduled maintenance interventions relative to the total number planned per year	X				X	
	Percent increase in ART cycles per million population per year		X				
	Percentage of accidents during handlings relative to the total number of ART procedures conducted in a given time period	X				X	
	Percentage of staff injuries in a given time period relative to the total number of ART procedures conducted					X	
	The existence of a website of the fertility clinic containing all the basic information, contracts and information about studies and FAQs at a certain moment in time		X				
	The provision of a clearly explained vision of the fertility clinic concerning ethical limitations of which at no time nor for no reason can be deviated at a certain moment in time			X			
	The provision of clearly described in- and exclusion criteria for MAR in the fertility clinic (among others taking into account the national legislation) at a certain moment in time			X			
	The provision of protocols that are in accordance with international guidelines/recommendations of care concerning equity and taking account of the universal needs at a certain moment in time			X			
	The provision of the offer to patients of psychosocial counseling at a certain moment in time				X		
	The provision of the use of an electronic patient record containing all relevant clinical information and allowing the extraction of letters and reports at a certain moment in time		X				
	The regular organization of a multidisciplinary meeting of the fertility clinic in which the psychosocial context of the patient can be discussed if necessary during a certain time period				X		
	The total number of FTE care providers relative to the total number of treated patients per type of care provider during a certain time period		X				

1PN, one pro-nucleus; ART, assisted reproductive technology; CPD, continuous professional development; C-LBR, cumulative live birth rate; CPR, clinical pregnancy rate; ED, embryo donation; ET, embryo transfer; FAQ, frequently asked question; FET, frozen embryo transfer; FTE, full-time equivalent; GIFT, gamete intrafallopian transfer; ICSI, intracytoplasmic sperm injection; IUI-H, intrauterine insemination with Husband sperm; IUI-D, intrauterine insemination with donor sperm; IVF, *in vitro* fertilization; KPI, key performance indicators; MII, metaphase II; MAR, medically assisted reproduction; OS, ovarian stimulation; OHSS, ovarian hyperstimulation syndrome; OPU, ovum pick up; PGD, pre-implantation genetic diagnosis; PGS, pre-implantation genetic screening; PGT, pre-implantation genetic testing; SIR, sustained implantation rate.

Most indicators were utilized as outcome indicators (69.72%) with fewer being process indicators (16.34%) or structural indicators (13.94%) (Table V). In terms of dimension, most indicators were efficiency (44.62%) and effectiveness (41.43%) indicators with fewer being safety, patient-centeredness, equity and timeliness indicators (Table V).

**Table V:** The relative landscape of types and dimensions of indicators.

Typology	Dimensions						
	Effectiveness	Efficiency	Equity	Patient-centeredness	Safety	Timeliness	
Outcome	87	82	0	0	6	0	175 (69.72%)
Process	10	14	1	3	6	7	41 (16.34%)
Structural	7	16	3	3	6	0	35 (13.94%)
	104 (41.43%)	112 (44.62%)	4 (1.59%)	6 (2.39%)	18 (7.17%)	7 (2.80%)	

### Responsibilities for data compilation

Fertility clinics shared data either manually through web-based applications or through an upload from a clinic’s electronic medical record system. Data from fertility clinics were sent to registries directly in 42 countries (48%) through national health authorities in 23 countries (26), medical organizations in 10 countries (11%), professional societies in 7 countries (8%) or based on personal initiative in 6 countries (7%). The reporting of data was either voluntary in 63 countries (72%) or compulsory in 25 countries (28%). The reporting of data was partial in 63 countries (72%) and complete in 25 countries (28%). Reporting for cycles or deliveries were based on individual cycles in 56 countries (64%) and on cumulative cycles in 32 countries (36%) (Table III).

### Purposes of indicators

Different organizations used the reported infertility and fertility care indicators for multiple reasons. The reported reasons for utilization were to monitor and to improve the quality of fertility care, to allow more accurate evaluation of practices, to enable evaluation of treatment outcomes, to allow ART outcomes to be monitored and compared and to assess the efficacy and safety of ART clinics. Furthermore, the indicators were used to benchmark institutions for targeting and evaluating improvement projects, for supporting accountability, regulations, and accreditation, and for assisting consumers’ choice of providers. Finally, indicators were utilized to facilitate evidence-based decision making, assist professionals to identify and subsequently target the domains of care in need of improvement, identify trends and areas where improvements can be made, make data available to researchers conducting important research, measure success strictly through quality of care, enable patients to make informed decisions on their treatment options, promote patient safety by disincentivizing risky practices (e.g. multiple embryo transfers), provide accurate access to health information, provide information and data to local, national and international stakeholders, disseminate reports of interest to the public, and enhance visibility and awareness of fertility care issues (Davies *et al.*, 2004; Rutstein and Shah, 2004; Malhotra *et al.*, 2013; Wilkinson *et al.*, 2017; Fauser, 2019; Jain *et al.*, 2019; Zahmatkeshan *et al.*, 2019a; Bai *et al.*, 2020; Centers for Disease Control and Prevention, 2020; De Geyter *et al.*, 2020; Dyer *et al.*, 2020a,b; Fischer and Scott, 2020; Lanes *et al.*, 2020; Newman *et al.*, 2020; Pirtea *et al.*, 2020; Sunderam *et al.*, 2020; Zegers-Hochschild *et al.*, 2020).

### Challenges in collecting data by healthcare facilities

Much of the data required for the reported indicators needs to be provided by fertility clinics, and this imposes several practical challenges. These practical challenges included the cost and time required to compile data when patients receive services across different sites, particularly if different record formats are used between those sites. The current use of paper for clinic records means that trained staff must manually abstract information. Further, clinic records often do not include clinic-specific patient selection practices, which is important since patient selection criteria affect the success rates. The loss to follow up of ART pregnancies significantly influences the ability to report and interpret delivery rates and the number of healthy babies born at term (the gold standard for measuring ART success). Finally, routinely collected records in some facilities may not include information regarding stratification by standard age groups and number of embryos transferred. The challenges identified in respect to national surveys included the possibility that survey results could be inaccurate due to vague or poorly worded questions, non-standardized procedures of survey administration and the potential risk of sampling or response biases (Davies *et al.*, 2004; Rutstein and Shah, 2004; Germond *et al.*, 2008; Mourad *et al.*, 2008; Breejen *et al.*, 2013; Dancet *et al.*, 2013; Malhotra *et al.*, 2013; Wilkinson *et al.*, 2017; Fauser, 2019; Jain *et al.*, 2019; Zahmatkeshan *et al.*, 2019a,b; Bai *et al.*, 2020; Centers for Disease Control and Prevention, 2020; De Geyter *et al.*, 2020; Dyer *et al.*, 2020a,b; Fischer and Scott, 2020; Lanes *et al.*, 2020; Newman *et al.*, 2020; Pirtea *et al.*, 2020; Sunderam *et al.*, 2020; Zegers-Hochschild *et al.*, 2020).

## Discussion

This systematic scoping review is the first to provide a broad overview of the landscape of the reported indicators of infertility and fertility care globally, and thus can act as the foundation for further work to select a core set of indicators through the identification of a consensus view across subject experts and stakeholders.

Overall, this review found that most indicators were outcome indicators with few structural and process indicators. Most indicators were effectiveness and efficiency indicators, while indicators of safety, patient-centeredness, equity and timeliness remain almost invisible. The scope of the current indicators indicates a predominant focus on clinical metrics, with substantial invisibility of non-clinical indicators and indicators outside the ART domain. These gaps need to be considered in further work of identifying a core set of indicators.

The finding that ART dominates the indicator landscape is obvious and explains the predominance of clinical over non-clinical indicators. It is notable that within the clinical domain, safety was less reported than effectiveness, which suggests that a successful treatment outcome (e.g. pregnancy rates) is often considered the golden standard in the evaluations of fertility treatments. Although recipients of care also highly value the ability of interventions to achieve live births (Scotland *et al.*, 2007; Palumbo *et al.*, 2011), it is essential to have indicators of minimizing potential harm (Dancet *et al.*, 2013).

Equitable access is a major topic in global reproductive health (Ombelet, 2011). It is alarming to observe that only 1.5% of the current landscape of indicators relate to equity.

This review demonstrates that many organizations and stakeholders collect a number of fertility-related indicators for their own planning, for benchmarking institutions, for targeting and evaluating improvement projects, for supporting accountability, regulations and accreditation and for assisting consumers’ choice of providers, as well as for reporting to different international agencies and donors. However, there is substantial variability in how different indicators are being collected, used or reported and in the responsible entities. Given this variability and the wide number of indicators, and in order to make appropriate comparisons, priority should be given to a subset of standardized indicators that provide the most crucial information for decision. Such prioritization should be based on agreed criteria.

Results of this review and the consultation emphasized the need to ensure that the indicators are well defined. Criteria can also be based on learning from other sectors in and beyond global health areas Examples of criteria used to select other indicators include: (i) an indicator must be relevant to the respective target; (ii) there must be an established methodology to measure it; (iii) data must be available from a wide range of countries to permit calculation of regional aggregates and time trends and (iv), a specified authority should assume responsibility to compile, estimate and release data (Barot *et al.*, 2015; United Nations, 2019).

In addition, an ideal indicator would have the following key characteristics: the indicator is based on agreed definitions and described exhaustively and exclusively; the indicator is highly or optimally specific and sensitive; the indicator is valid and reliable; the indicator discriminates well; the indicator relates to clearly identifiable events for the user; the indicator permits useful comparisons and the indicator is evidence-based (Mainz, 2003). These considerations will be useful in identifying a core set of clinical and non-clinical indicators, for fertility care, some of which may not be in routine use. Given the challenges in collecting and compiling indicator data, consulting with multiple stakeholders will be essential in identifying practical and implementable indicators.

Having a priority/core and expanded set of indicators that includes both clinical and non-clinical metrics will not limit the choice of indicators that may be used globally. Indeed, stakeholders and clinics can utilize relevant indicators to assess their multidimensional quality of care. A reasonable strategy is to select a package of indicators that meet the needs of people with infertility; sometimes these will be structure or process measures, and sometimes they will be outcomes measures, and as noted in this review, they may also need to include non-clinical domains of fertility care.

This review has some limitations. Although a comprehensive search was made for existing literature regardless of date, language and peer review status, it is possible that some data were not captured. In addition, we included all studies regardless of their quality, as our intention was to assess the extent of the available literature, to organize it into typologies and to highlight gaps. Despite these limitations, this review provides useful data for future research and work related to global indicators for fertility care.

Indicators of infertility and fertility care exist in the literature, mainly as outcomes indicators of effectiveness and efficiency dimensions. Organizations can utilize the available indicators that best suit the needs of individuals and communities. Literature on the safety, patient-centeredness, equity and timeliness of infertility care was limited. A gap was identified in relation to non-clinical indicators. The results of this scoping review serve as the basis to develop a core set of indicators using a consensus process that involves multiple stakeholders.

## Supplementary data

Supplementary data are available at *Human Reproduction Open* online.

## Supplementary Material

hoac047_Supplementary_Data

## Data Availability

All data generated or analyzed during this study are included in this published article and its supplementary information files.
