# Supplementary material for: Indicators of infertility and fertility care: a systematic scoping review
Source: Hum Reprod Open. 2022 Oct 13;2022(4):hoac047. doi: 10.1093/hropen/hoac047 (PMC9632452; doi:10.1093/hropen/hoac047)
Supplement: hoac047_Supplementary_Data [file hoac047_supplementary_data.docx]

## Supplementary Table SI: Search strategy

| Bibliographic database | Search query |
| --- | --- |
| PubMed | Humans [Mesh] AND (Infertility [Mesh] OR assisted reproduct* [tw] OR infertility [tw] OR subfertility [tw]) AND (Patient Care/standards [Mesh] OR Health Care Quality Indicators [Mesh] OR indicator* [tw]) |
| MEDLINE | exp Humans/ AND (exp Infertility/ OR assisted reproduct*.mp. OR infertility.mp. OR subfertility.mp.) AND (exp Patient Care/standards/ OR exp Quality Indicators, Health Care/ OR indicator*.mp.) |
| Web of Science | Humans AND (Infertility OR “assisted reproduct*” OR infertility OR subfertility) AND (“Patient Care/standards” OR “Quality Indicators, Health Care” OR indicator*) |
| CINAHL | (MH “Humans+”) AND ((MH “Infertility+”) OR “assisted reproduct*” OR infertility OR subfertility) AND ((MH “Patient Care/standards+”) OR (MH “Quality Indicators, Health Care+”) OR indicator*) |
| Scopus | INDEXTERMS(“Humans”) AND (INDEXTERMS(“Infertility”) OR TITLE-ABS-KEY(“assisted reproduct*”) OR TITLE-ABS-KEY(“infertility”) OR TITLE-ABS-KEY(“subfertility”)) AND (INDEXTERMS(“Patient Care/standards”) OR INDEXTERMS(“Quality Indicators, Health Care”) OR TITLE-ABS-KEY(“indicator*”)) |

## Supplementary Table SII: Data items

| Data extracted |
| --- |
| Indicator |
| Dimension |
| Type |
| Country |
| Requirements |
| Responsibility |
| Reporting methods |
| Proportion of Clinics reporting data |
| Limitations |

**Supplementary Table SIII: Quality assessment of included primary reports**

| ID | Year | Is there a clear research question | Do the collected data allow to address the research question? | Is the sampling strategy relevant to address the research question? | Is the sample representative of the target population? | Are the measurements appropriate? | Is the risk of nonresponse bias low? | Overall quality |
| --- | --- | --- | --- | --- | --- | --- | --- | --- |
| Adamson | 2018 | Yes | Yes | Yes | Yes | Yes | Yes | High |
| AFSR | 1993 | Yes | Yes | Yes | Yes | Yes | Yes | High |
| AFSR | 1994 | Yes | Yes | Yes | Yes | Yes | Yes | High |
| AFSR | 1996 | Yes | Yes | Yes | Yes | Yes | Yes | High |
| AFSR | 2002 | Yes | Yes | Yes | Yes | Yes | Yes | High |
| AFSR | 2007 | Yes | Yes | Yes | Yes | Yes | Yes | High |
| Dancet | 2013 | Yes | Yes | Yes | Yes | Yes | Yes | High |
| Davies | 2004 | Yes | Yes | Yes | Yes | Yes | Yes | High |
| De Geyter | 2020 | Yes | Yes | Yes | Yes | Yes | Yes | High |
| De Neubourg | 2016 | Yes | Yes | Yes | Yes | Yes | Yes | High |
| den Breejen | 2013 | Yes | Yes | Yes | Yes | Yes | Yes | High |
| Dyer | 2016 | Yes | Yes | Yes | Yes | Yes | Yes | High |
| Dyer | 2020 | Yes | Yes | Yes | Yes | Yes | No | Some concerns |
| Ferraretti | 2013 | Yes | Yes | Yes | Yes | Yes | Yes | High |
| Germond | 2008 | Yes | Yes | Yes | Yes | Yes | Yes | High |
| Jain | 2019 | Yes | Yes | Yes | Yes | Yes | Yes | High |
| Kupka | 2014 | Yes | Yes | Yes | Yes | Yes | Yes | High |
| Lanes | 2020 | Yes | Yes | Yes | Yes | Yes | Yes | High |
| Malhotra | 2013 | Yes | Yes | Yes | Yes | Yes | No | Some concerns |
| Mansour | 2014 | Yes | Yes | Yes | Yes | Yes | Yes | High |
| Mourad | 2008 | Yes | Yes | Yes | Yes | Yes | Yes | High |
| Newman | 2020 | Yes | Yes | Yes | Yes | Yes | Yes | High |
| Sullivan | 2013 | Yes | Yes | Yes | Yes | Yes | Yes | High |
| Sunderam | 2012 | Yes | Yes | Yes | Yes | Yes | Yes | High |
| Sunderam | 2013 | Yes | Yes | Yes | Yes | Yes | Yes | High |
| Sunderam | 2014 | Yes | Yes | Yes | Yes | Yes | Yes | High |
| Sunderam | 2015 | Yes | Yes | Yes | Yes | Yes | Yes | High |
| Sunderam | 2015 | Yes | Yes | Yes | Yes | Yes | Yes | High |
| Sunderam | 2017 | Yes | Yes | Yes | Yes | Yes | Yes | High |
| Sunderam | 2018 | Yes | Yes | Yes | Yes | Yes | Yes | High |
| Sunderam | 2019 | Yes | Yes | Yes | Yes | Yes | Yes | High |
| Wilkinson | 2017 | Yes | Yes | Yes | Yes | Yes | Yes | High |
| Wright | 2003 | Yes | Yes | Yes | Yes | Yes | Yes | High |
| Wright | 2004 | Yes | Yes | Yes | Yes | Yes | Yes | High |
| Zahmatkeshan | 2019 | Yes | Yes | Yes | Yes | Yes | Yes | High |
| Zahmatkeshan | 2019 | Yes | Yes | Yes | Yes | Yes | Yes | High |
| Zegers-Hochschild | 2014 | Yes | Yes | Yes | Yes | Yes | Yes | High |
| Zegers-Hochschild | 2020 | Yes | Yes | Yes | Yes | Yes | No | Some concerns |
